# Design and evaluation of a ball spline wasp-inspired needle

**DOI:** 10.3389/fbioe.2024.1468605

**Published:** 2024-11-28

**Authors:** Jette Bloemberg, Zola Fung-A-Jou, Paul Breedveld, Aimée Sakes

**Affiliations:** Department of BioMechanical Engineering, Faculty of Mechanical Engineering, Delft University of Technology, Delft, Netherlands

**Keywords:** bio-inspiration, ovipositor, needle, ball spline, medical device design

## Abstract

In percutaneous interventions, needles are used to reach target locations inside the body. However, when the needle is pushed through the tissue, forces arise at the needle tip and along the needle body, making the needle prone to buckling. Recently, needles that prevent buckling inspired by the ovipositor of female parasitic wasps have been developed. Building on these needle designs, this study proposes a manual actuation unit that allows the operator to drive the wasp-inspired needle through stationary tissue. The needle consists of six 0.3-mm spring steel wires, of which one is advanced while the others are retracted. The advancing needle segment has to overcome a cutting and friction force while the retracting ones experience a friction force in the opposite direction. The actuation unit moves the needle segments in the required sequence using a low-friction ball spline mechanism. The moving components of the needle have low inertia, and its connection to the actuation unit using a ball spline introduces a small friction force, generating a small push force on the needle that facilitates the needle’s propulsion into tissue while preventing needle buckling. Experimental testing evaluated the needle’s ability to move through stationary 15-wt% gelatin tissue phantoms for different actuation velocities. It was found that the needle moved through the tissue phantoms with mean slip ratios of 0.35, 0.31, and 0.29 for actuation velocities of π, 2π, and 3π rad/s, respectively. Furthermore, evaluation in 15-wt%, 10-wt%, and 5-wt% gelatin tissue phantoms showed that decreasing the gelatin concentration decreased the mean slip ratios from 0.35 to 0.19 and 0.18, respectively. The needle actuation system design is a step forward in developing a wasp-inspired needle for percutaneous procedures that prevents buckling.

## 1 Introduction

### 1.1 Wasp-inspired needles

Percutaneous interventions are used to create a passageway to a target location inside the body, inject or extract fluids, extract tissue samples, and position instruments such as radioactive seeds and optical fibers at precise locations in deep-seated tissue structures, such as the prostate gland. Biopsies, regional anesthesia, brachytherapy, and focal laser ablation rely on needles to perform the procedure. When a clinician inserts the needle by pushing it through the tissue, forces arise at the needle tip and along the needle body (Okamura et al., 2004). When the axial force on the needle exceeds the needle’s critical load, the needle will deflect laterally due to buckling (Sakes et al., 2016). The lateral deflection might cause tissue damage and lead to poor control of the needle trajectory, potentially decreasing precision (Abolhassani et al., 2007a; Abolhassani et al., 2007b). Buckling phenomena are typically not a part of the needle trajectory plan, yet they are reported in deep-tissue needle insertion experiments (Reed et al., 2011; Rucker et al., 2013).

Buckling is a failure mode where an equilibrium configuration becomes unstable under excessive compression, leading to a sudden lateral deflection and potential obstruction of the lumen of the needle. Slender ideal columns under compression are subject to Euler buckling. The critical Euler buckling load can be calculated as Equation 1.

$$F_{\text{cr}}=\frac{\pi^{2}EI}{{\left(KL\right)}^{2}}$$ (1)

Where E is the Young’s Modulus of the needle (N mm^-2^), I the second moment of area of the cross-section of the needle (mm^4^), K a coefficient that takes into account the end conditions of the needle $\left(..\right)$ , and L the unsupported length of the needle (mm) (Euler, 1757). The extended critical Euler buckling load Equation 2 defines the critical load for a needle inserted into a substrate.

$$F_{\text{cr}\;\text{ext}}=\frac{\pi^{2}EI}{{\left(KL\right)}^{2}}+\frac{\mu L^{2}}{\pi^{2}}$$ (2)

Where μ is the spring stiffness of the substrate (N mm^-2^) (Chen and Atsuta, 2007; He et al., 2008). To prevent buckling during needle insertion, the insertion force ( $F_{\text{in}}$ ) applied to the needle inside the substrate should remain below the critical load of the needle (**F**_cr ext_) (Sakes et al., 2016).

**FIGURE 1:**
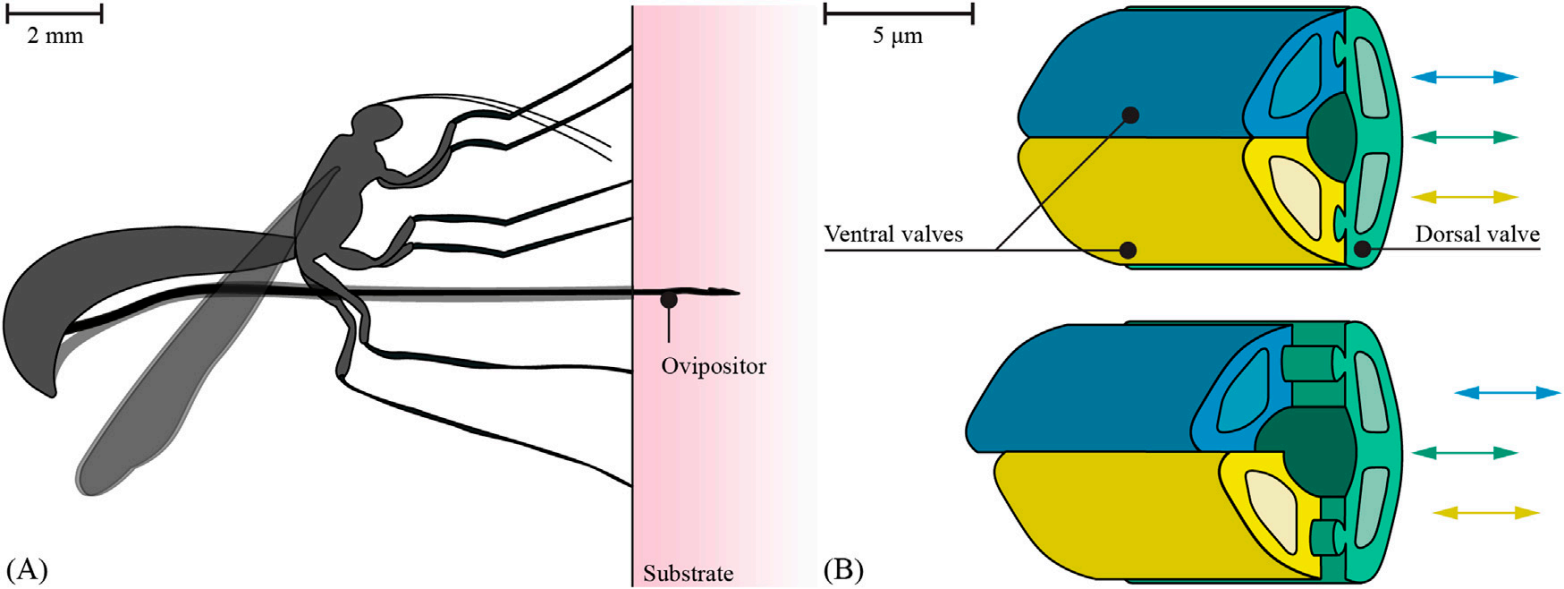
Visualization of the motion sequence of the ovipositor of a parasitic wasp. **(A)** The parasitic wasp uses its ovipositor to lay eggs in a substrate. **(B)** The ovipositor consists of three parallel valves (green, yellow, and blue) that can move reciprocally (based on Cerkvenik et al. (2017)).

To prevent needle buckling and reduce tissue damage during needle insertion, needles inspired by the ovipositor of the female parasitic wasp have been developed that can be advanced through the tissue without an external push force (Frasson et al., 2010; Leibinger et al., 2016; Scali et al., 2017a; Scali et al., 2019). Female parasitic wasps use their ovipositor to lay eggs within hosts, which may hide in compact substrates such as wood (Quicke and Fitton, 1995). The ovipositor of the parasitic wasp *Diachasmimorpha longicaudata* (Hymenoptera: Braconidae) is characterized by its considerable length (5.7 ± 0.6 mm) (Leyva et al., 1991) and small diameter (30–50 µm) (Cerkvenik et al., 2019). It consists of three slender, parallel segments, called valves (Cerkvenik et al., 2017), which reciprocate through advancing and retracting movements with respect to each other (Figure 1). The reciprocal advancing and retracting forces create a net insertion force near zero, facilitating self-propulsion within a substrate without buckling.

The wasp-inspired needles use multiple mechanisms to prevent buckling. Firstly, the needle consists of multiple parallel needle segments that employ an advancing-retraction mechanism, where one needle segment is advanced while the other needle segments are retracted. The advantage of this advancing-retraction mechanism compared to pushing the entire needle through the tissue is the decreased axial load applied to the needle. The friction and cutting forces of the advancing needle segment inside the substrate are (partially) counterbalanced by the friction force in the opposite direction of the retracted needle segments, thereby decreasing the insertion force required to insert the needle into the substrate, thus decreasing $F_{\text{in}}$ . Secondly, the needle segments move incrementally forwards, traversing over a short distance per needle movement, the unsupported length L inside the substrate is therefore kept low and independent of insertion depth, thereby increasing $F_{\text{cr}\;\text{ext}}$ .

### 1.2 Problem definition

Inserting a needle into tissue results in forces acting on the needle by the surrounding tissue. Okamura et al. (2004) demonstrated that these forces are the sum of the surface stiffness force $\left(F_{\text{stiff}}\right)$ due to the needle puncturing the skin or surface membrane until the moment of puncture (Heverly et al., 2005), cutting force $\left(F_{\text{cut}}\right)$ due to the plastic deformation and tissue stiffness experienced at the tip of the needle (Ng et al., 2013), and friction force $\left(F_{\text{fric}}\right)$ along the needle length inside the tissue due to Coulomb friction, adhesive friction, and viscous friction (Kataoka et al., 2002). The motion of the wasp-inspired needle is initiated after puncturing the skin or membrane and is accomplished by parallel needle segments, more specifically by a advancing segments that move forward and r retracting segments that move backward. The advancing segments experience a cutting and friction force, whereas the stiffness force is zero when assuming homogeneous tissue after puncturing. The retracting segments experience a friction force in the opposite direction compared to the advancing segments (Scali, 2020). Using the conditions for the self-propulsion of the wasp-inspired needle and Newton’s second law, we get:

$$\sum_{i=1}^{a}\left({F_{\text{stiff},i}+F}_{\text{fric},i}+F_{\text{cut},i}\right)\;{+F}_{\text{fric},m}+m_{m}a_{m}\leq \sum_{j=1}^{r}F_{\text{fric},j}$$ (3)

Where $F_{\text{stiff},i}$ is the stiffness force on the tip of the advancing needle segment, which is assumed to be zero, $F_{\text{fric},i}$ is the friction force along the advancing needle segment, $F_{\text{cut},i}$ is the cutting force on the tip of the advancing needle segment, and $F_{\text{fric},j}$ is the friction force along the retracting needle segments, which works in the opposite direction as the friction force of the advancing needle segments. Furthermore, ${\;F}_{\text{fric},m}$ is the friction force of the moving components, and $m_{m}$ and $a_{m}$ their mass and acceleration, respectively. The needle self-propels through the tissue if the friction force generated by the retracting needle segments overcomes the friction and cutting force of the advancing needle segments and the friction force and inertia of the moving components. This way, the retracting needle segments remain stationary with respect to the tissue, whilst the advancing segments move forward into the tissue.

**FIGURE 2:**
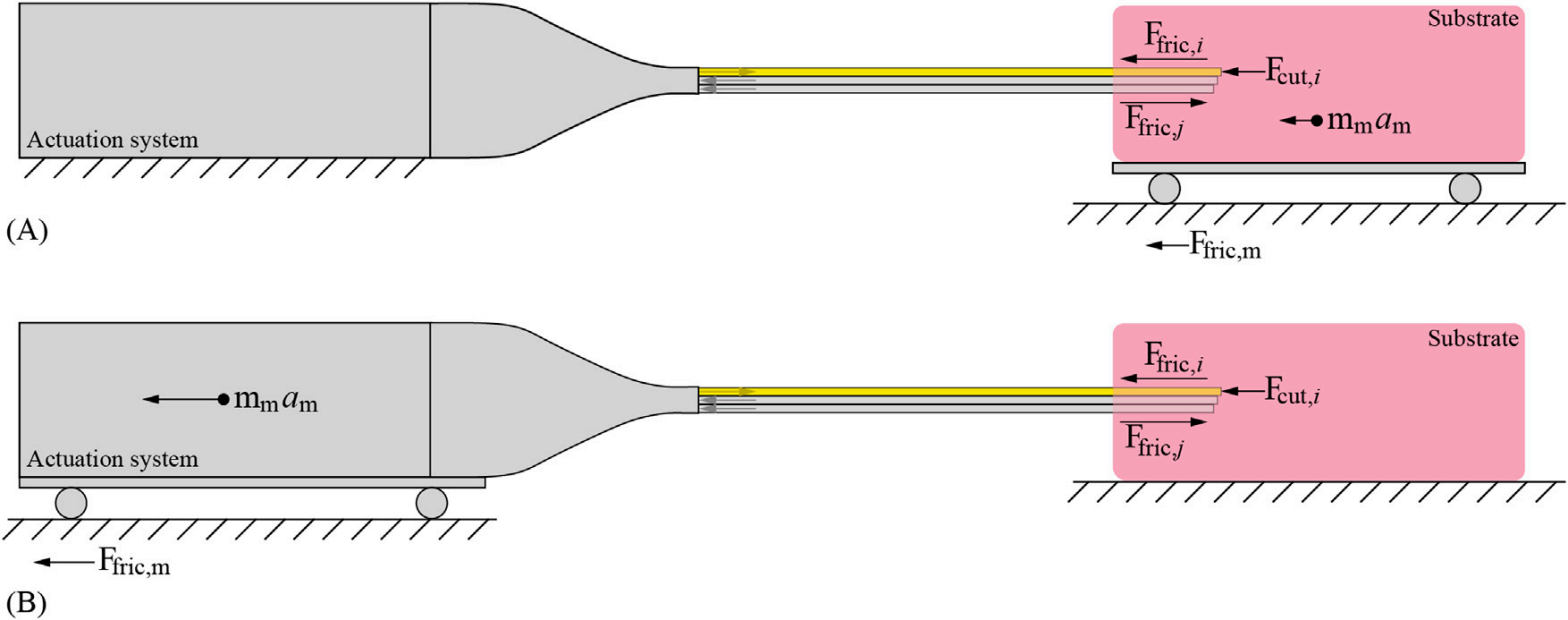
Visualization of the motion sequence of the wasp-inspired needles. During the motion, one needle segment (in yellow) moves forward over the stroke distance while the others (in gray) move slowly backward. $F_{\text{fric},i}$ is the friction force along the advancing needle segment, $F_{\text{cut},i}$ is the cutting force on the tip of the advancing needle segment, and $F_{\text{fric},j}$ is the friction force along the retracting needle segments, which works in the opposite direction as the friction force of the advancing needle segments. ${\;F}_{\text{fric},m}$ is the friction force of the moving components, and $m_{m}$ and $a_{m}$ their mass and acceleration, respectively. **(A)** Configuration where the actuation system remains stationary while the substrate moves towards the needle. **(B)** Configuration where the substrate remains stationary while the actuation system and the needle move towards the substrate.

For the self-propelled motion to be effective, the friction force and inertia of the moving components should be negligibly small. Current wasp-inspired self-propelled needles were constructed in either of the following configurations. In the first configuration, the actuation system remained stationary while the substrate was placed on a low-weight, low-friction support structure that could move towards the needle following the pace of the self-propelled motion of the needle (Scali et al., 2017a; Scali et al., 2019; Bloemberg et al., 2022; Scali et al., 2017b; Parittotokkaporn et al., 2008) (Figure 2A). However, in clinical practice, the needle must self-propel inside the patient while the substrate, and thus the patient, remains in place. In the second configuration, the substrate remained stationary while the actuation system and the needle were placed on a low friction cart that could move towards the substrate following the pace of the self-propelled motion of the needle (Sprang et al., 2016) (Figure 2B). A disadvantage of the second configuration is that the inertia and friction of the needle, including its actuation system, oppose the self-propulsion of the needle.

### 1.3 Goal of this study

Current prototypes of wasp-inspired self-propelled needles are designed as experimental setups in which either the needle and actuation system remained stationary while the tissue (phantom) was moved towards them (Scali et al., 2017a; Scali et al., 2019; Bloemberg et al., 2022; Scali et al., 2017b; Parittotokkaporn et al., 2008), or the tissue remained stationary while the needle and actuation system could move (Sprang et al., 2016). Both options are not optimal for integration in clinical practice and the performance of the needle due to the inertia that negatively affects the needle’s self-propelled motion. In clinical practice, the needle will have to self-propel inside the patient while both the actuation system and the tissue remain in place. Therefore, this study aims to design a stationary manual actuation system for a needle that uses the self-propelling principle of the parasitic wasp and can travel through stationary tissue (phantoms). Specifically, the moving components that connect the needle segments to the actuation system should have negligible influence on the self-propelled motion of the needle. Therefore, the moving components that connect the needle segments to the actuation system should have low inertia and the connection itself should introduce friction forces near zero.

## 2 Design

### 2.1 Needle

**FIGURE 3:**
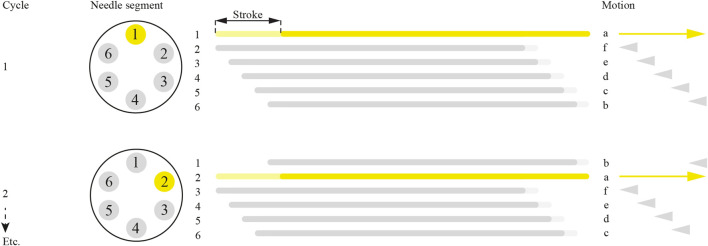
Visualization of the motion sequence of the needle segments of the wasp-inspired needle. During the motion, one needle segment (in yellow) moves forward over the stroke distance while the other needle segments (in gray) move slowly backward over one-fifth of the stroke distance in a consecutive manner.

The complete design, called the Splinositor, consists of a needle and an actuation system. The needle consists of six parallel needle segments following the designs by Scali et al. (2019), Bloemberg et al. (2022). The needle self-propels through the substrate by a sequential translation of the six needle segments in six steps per cycle (Figure 3). During every step of the cycle, one needle segment moves forward over a specified distance called the “stroke”, while the other five needle segments move slowly backward over one-fifth of the stroke distance, similar to the needle design by Bloemberg et al. (2022). Every needle segment is moved forward over the stroke distance once during one cycle.

When the distal ends of the needle segments are positioned inside tissue, they experience friction forces with the surrounding tissue. When the self-propelling principle, as in Equation 3 holds, the retracting needle segments remain stationary with respect to the tissue, whilst the advancing segment moves forward into the tissue. By repeating the actuation cycle, the needle as a whole advances in the tissue.

### 2.2 Actuation system

We opted for a manually controlled actuation system that allows the operator to drive the needle directly and intuitively using a continuous manual rotation around the axis of needle insertion, i.e., the horizontal *y*-direction. By using the manual actuation force solely for an input rotation, the operator cannot apply an external insertion force to the needle to push the needle into the tissue. Consequently, the operator cannot interfere with the self-propelled motion of the needle segments. The actuation system converts the input rotation into a sequential translation of the six needle segments in the required order and over the required stroke distance while minimizing the number of components that travel with the needle segments.

**FIGURE 4:**
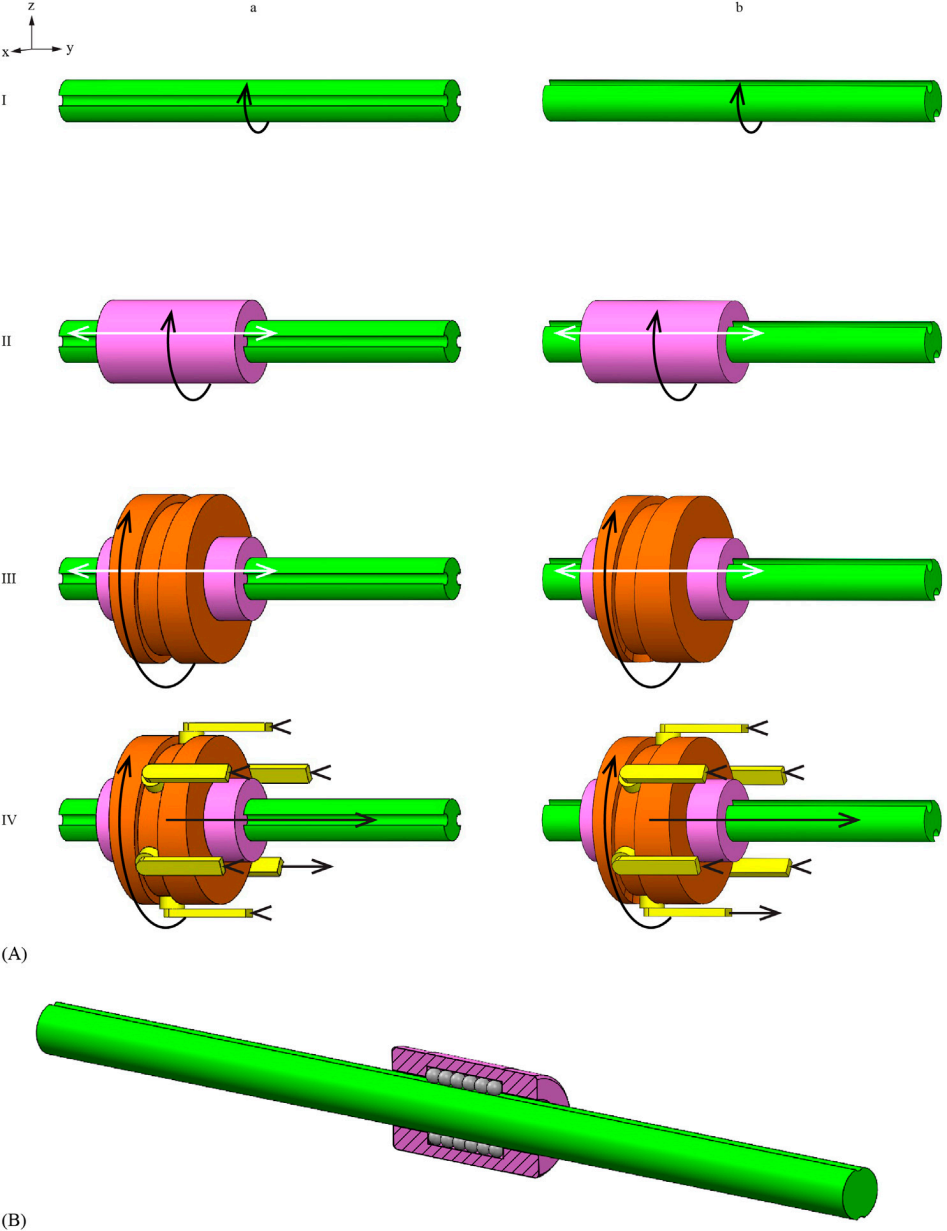
Schematic representation of the actuation system motion mechanism. **(A)** The actuation system includes the drive cylinder (green), follower cylinder (pink), cam (orange), and cam followers (yellow) that contain the needle segments. Columns a and b show two phases in the motion cycle (i.e., 60° difference) and Rows I-IV show the different layers of the actuation system. **(B)** The ball spline mechanism, including the drive cylinder (green) with grooves, cross-section of the ball spline follower cylinder (pink) with grooves, and ball bearings (gray) rolling within the grooves.

To explain the working principle of our actuation system, the mechanism is simplified and visualized in a schematic illustration in Figure 4A, where Columns a and b show two phases in the motion cycle (i.e., 60° difference) and Rows I-IV show the different layers of the actuation system. The input motion is a rotation of the drive cylinder (in green) around the *y*-axis. The drive cylinder contains horizontal grooves. A follower cylinder (in pink) contains rims that fit in the drive cylinder’s grooves. Hence, the grooves and rims transmit the rotation around the *y*-axis to the follower cylinder while enabling a translation in the *y*-direction of the follower cylinder relative to the drive cylinder. Around the follower cylinder, a cam (in orange) is positioned containing a V-shaped slot, in which six cam followers (in yellow) can slide. The motion of the cam followers was restricted to solely a translation in the *y*-direction, driven by the motion of the V-shaped slot. The asymmetric shape of the V-shape in the slot causes one cam follower to move in the positive *y*-direction while the other cam followers move in the negative *y*-direction.

To minimize the friction force introduced by the connection of the needle to the actuation system, we implemented a ball spline mechanism (Figure 4B). The ball spline incorporates balls within the grooves in both the drive and follower cylinder, facilitating linear rolling translation of the follower cylinder relative to the drive cylinder while simultaneously transmitting rotation. The components of the actuation system that move with the needle segments include the follower cylinder, ball bearings of the ball spline mechanism, cam, and cam followers that house the needle segments. Because of the low-friction ball spline mechanism, the moving components of the actuation system introduce a friction force near zero.

### 2.3 Final design

**FIGURE 5:**
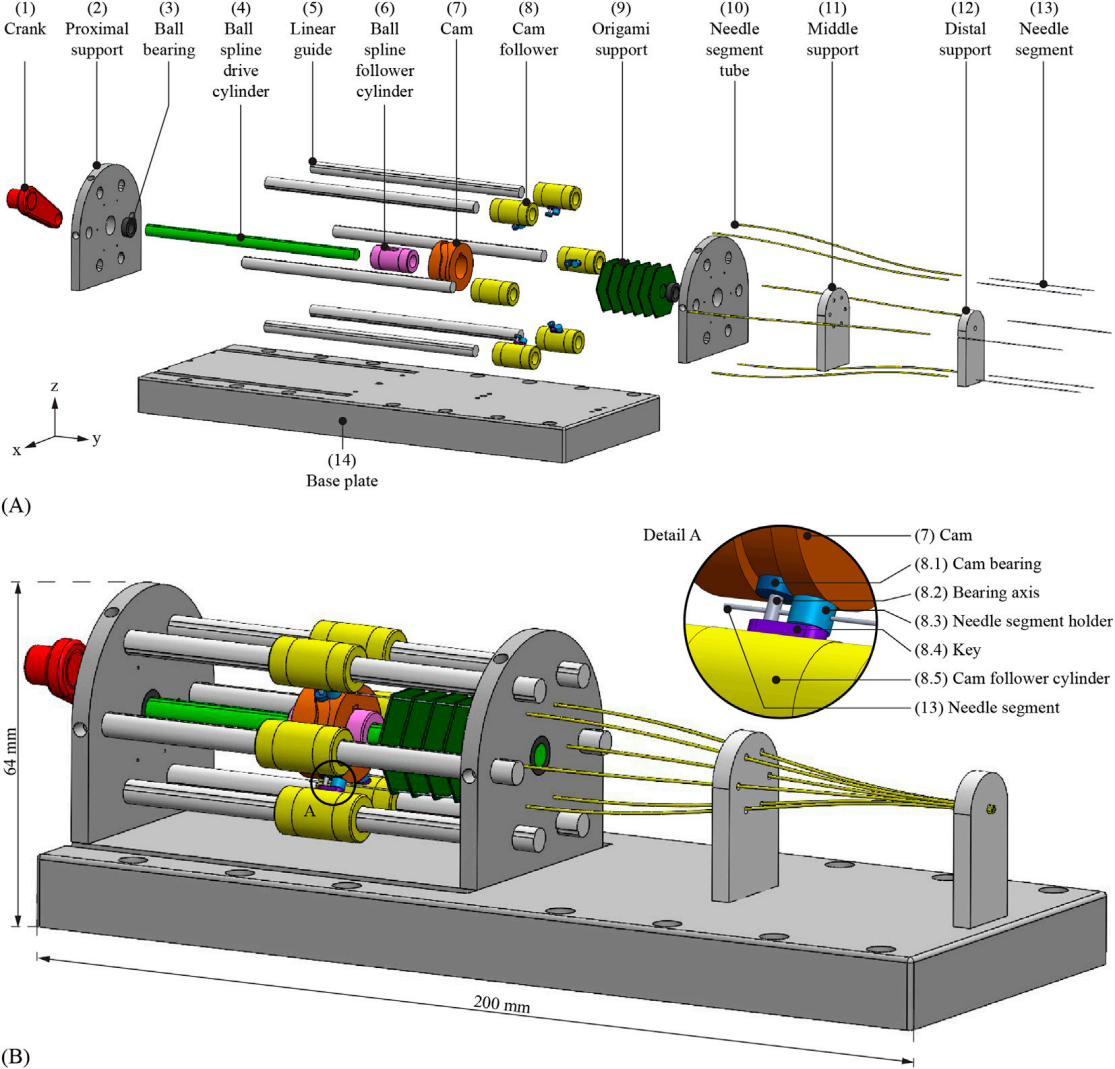
Schematic representation of the complete design of the Splinositor. **(A)** Exploded view. Crank (1), proximal support (2), ball bearing (3), drive cylinder of the ball spline (4), linear guide axis (5), follower cylinder of the ball spline (6), cam (7), cam follower (8), origami support structure (9), needle segment tube (10), middle support (11), distal support (12), needle segment (13), base plate (14). **(B)** Complete design assembly. The cam follower (8) that rolls within the cam (7) consists of the cam bearing (8.1), bearing axis (8.2), needle segment holder (8.3), key (8.4), and cam follower cylinder (8.5).

We used Solidworks (Dassault Systems Solidworks Corporation; Waltham, MA, United States) as Computer-Aided Design (CAD) software to design the Splinositor (Figure 5). To facilitate the manual actuation of the drive cylinder, we added a crank to the actuation mechanism that transmits the input rotation to the drive cylinder and the cam. The height of the cam track dictates a 4-mm stroke in the positive *y*-direction for the cam followers over a 60° rotation of the cam. During the following 300° rotation, the cam track dictates a 4-mm stroke in the negative *y*-direction. We chose off-the-shelf ball bearings as followers of the cam. Each cam follower contains a bearing axis, to attach it to a key, which was attached to a cam follower cylinder of a linear guide axis. A needle segment holder was also attached to the key. The six linear guides restrict the motion of the needle segment holders to a translation along the *y*-axis.

To prevent buckling of the needle segments before they enter the needle segment tubes, the needle segments require a support structure. We needed a movable support structure capable of translating along with the cam, as the unsupported needle length decreases when the needle segments propel in the tissue. We chose to implement an origami structure, i.e., a tube derived from the Miura-origami (Miura-ori) pattern (Wu et al., 2020), which is stiff in the *z*-direction but flexible in the *y*-direction. When the cam moves in the positive *y*-direction, the Miura-ori tube contracts. The Miura-ori tube contains small holes through which the needle segments pass, guiding and supporting the needle segments’ movement along the *y*-axis while avoiding buckling.

Through the actuation system, the needle segments run at a larger diameter than at the needle tip. To guide the needle segments smoothly from the actuation system to the needle tip, needle segment tubes with an S-curve were used. The S-curved needle segment tubes gently decrease the distance between the needle segments by guiding them smoothly through the S-shaped needle segment tubes from the actuation system to the needle tip. These tubes provide continuous support to the needle segments to avoid buckling, while allowing them to move along the *y*-axis freely.

### 2.4 Prototype

**FIGURE 6:**
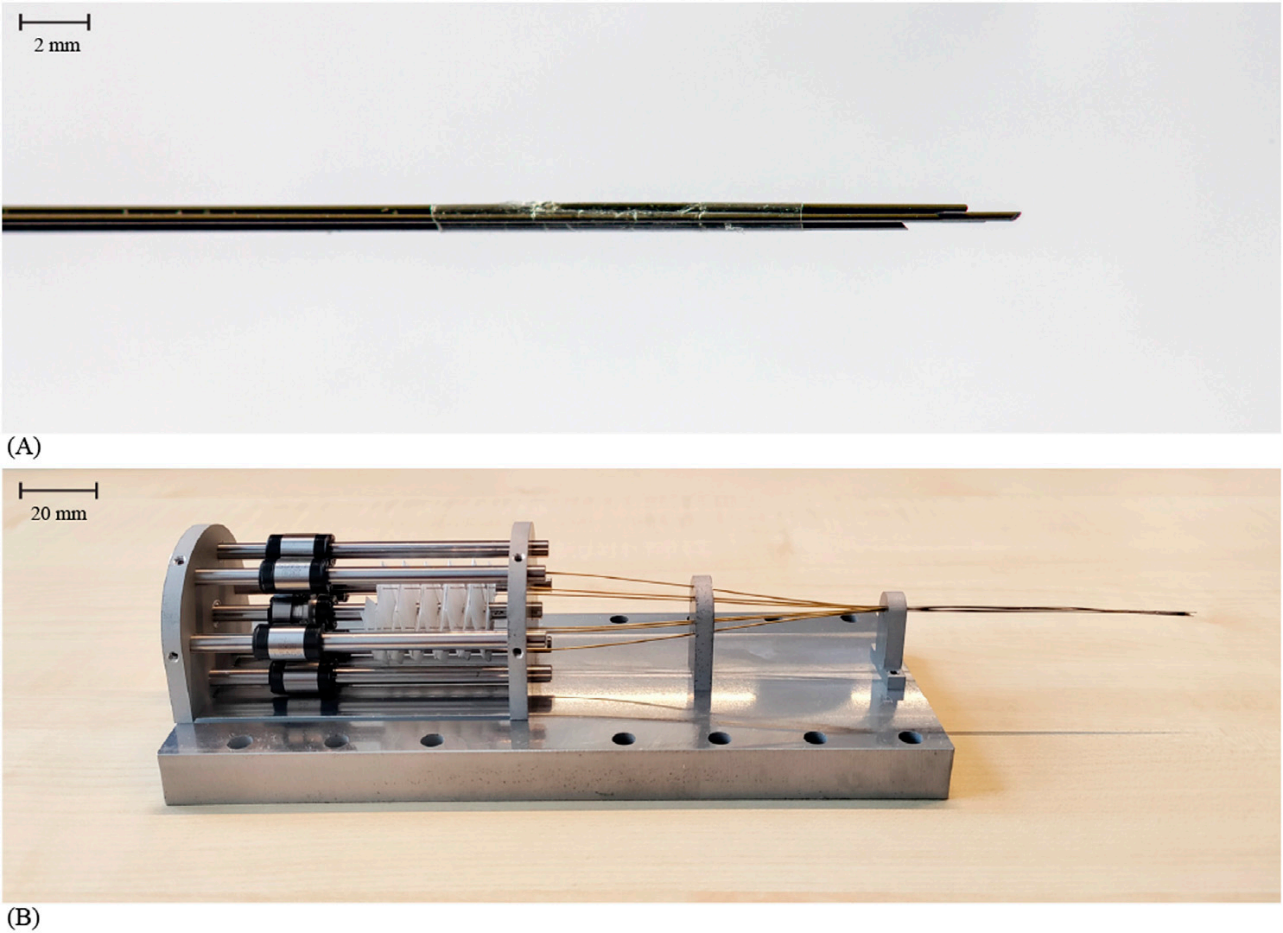
Final Splinositor prototype. **(A)** Close-up of the needle tip consisting of six sharpened spring steel rods held together by a heat shrink tube (Nordson Medical Corp.) glued to one of the six rods. **(B)** Assembled Splinositor prototype.

The needle in this study consists of six spring steel rods, i.e., the needle segments, with a diameter of 0.3 mm and a length of 230 mm (Figure 6A). The tips of the needle segments were sharpened to an angle of 20° with wire Electrical Discharge Machining (EDM). The needle segments were held together at the tip using a 10-mm long heat shrink tube (*1030352*, Nordson Medical Corp., Westlake, OH, United States). This tube was employed to limit the needle segments from diverging while only minimally increasing the needle diameter. To maintain its position at the needle tip, the heat shrink tube was glued to one of the needle segments using *Pattex Gold Gel 1432562* (Pattex, Henkel AG and Co., Düsseldorf, Germany). The remaining needle segments can move freely back and forth through the heat shrink tube. The resulting total diameter of the needle, including the heat shrink tube, is 0.99 mm.

Figure 6B shows the assembled prototype. The crank was produced using three-dimensional (3D) printing, specifically the fused deposition technology in polylactic acid (PLA) on an Ultimaker 3 printer. The ball splines used in the core mechanism of the actuation system (i.e., the drive cylinder) and the linear guides are stainless steel *Ball Splines LSAG10C1R200* (IKO Nippon Thompson Co. Ltd., Tokyo, Japan). The drive cylinder, with a length of 100 mm, facilitates a 100-mm travel distance of the needle inside the tissue. The central ball spline was positioned between two stainless steel *Deep Groove Ball Bearings DDL-850ZZMTHA1P13LY121* (MinebeaMitsumi Inc., Tokyo, Japan). The cam was milled out of 7075-T6 aluminum. The six cam bearings are stainless steel *Deep Groove Ball Bearings DDL-310HA1P25LO1* (MinebeaMitsumi Inc., Tokyo, Japan). The bearing axes are 125–245 HV30 steel *Cylindrical Pens 2338H8100010005* (Bossard, Zug, Switzerland). The aluminum needle segment holders and keys were produced using wire EDM. The proximal, middle, and distal supports form the actuation system’s support blocks, and were made from 7075-T6 aluminum using wire EDM. The support blocks were attached to the aluminum base plate that was also produced using wire EDM. The Miura-ori tube was cut and folded out of lightweight drawing paper. The needle segment tubes (inner diameter 0.4 mm, outer diameter 0.6 mm) were constructed from brass due to the advantageous low coefficient of friction between brass and the steel needle segments.

## 3 Proof-of-principle experiment

### 3.1 Experimental goal and variables

To evaluate the performance of the Splinositor under controlled conditions, we performed two experiments in gelatin phantoms. The experiments aimed to investigate the performance behavior of the needle actuated (1) for different actuation velocities and (2) inside different gelatin phantoms, both in terms of the slip of the needle with respect to the gelatin phantom. More specifically, we calculated the slip ratio of the needle while it advanced through the phantom, using Equation 4.

$$s_{\text{ratio}}=1-\left(\frac{d_{m}}{d_{t}}\right)$$ (4)

Where $d_{m}$ and $d_{t}$ are the measured and theoretical maximum traveled distance, respectively. The measured traveled distance is the difference in the position of the needle tip we measured in the video footage before and after needle actuation. The theoretical maximum traveled distance depends on the motion sequence (5:1), the stroke distance ( S ), which was 4 mm by design, and the number of actuation cycles ( C ) and was calculated using Equation 5.

$$d_{t}=\frac{6}{5}SC$$ (5)

The first experiment aimed to find the most efficient technical configuration of the Splinositor inside gelatin phantoms with a concentration of 15% weight (wt) powder in water. The independent variables were the mobility of the ball spline and the actuation speed ( ω ) of the needle. To investigate the effect of the central ball spline, we evaluated the prototype in two conditions in which:(1) the central ball spline was fixed to constrain its translation in the *y*-direction while allowing rotation around the *y*-axis, while the gelatin sample was placed on a low-friction cart, and (2) the central ball spline was able to move like intended (i.e., mobile) and the gelatin sample remained stationary. To investigate the effect of the actuation angular velocity ( ω ), ω was set at π, 2π, or 3π rad/s using a metronome.

The second experiment aimed to investigate the effect of the stiffness of the gelatin phantom on the Splinositor performance for the configuration with a mobile ball spline and an actuation velocity of π rad/s. The independent variable was the concentration of gelatin powder in the phantoms, which was set at 5 wt%, 10 wt%, and 15 wt%. These concentrations lead to gelatin samples with moduli of elasticity of approximately 5.3, 17, and 31 kPa (Scali et al., 2019), respectively. The 5-wt% gelatin phantom approximates soft tissue such as healthy liver tissue (<6 kPa) (Mueller and Sandrin, 2010). The 10-wt% gelatin phantom approximates tissue with an intermediate stiffness such as muscle tissue (12–32 kPa) (Kot et al., 2012) and healthy prostate tissue (16 kPa) (Zhang et al., 2008). The 15-wt% gelatin phantom approximates stiff tissue such as cancerous prostate tissue (40 kPa) (Zhang et al., 2008; Barr et al., 2012).

In both experiments, the dependent variable was the slip ratio ( $s_{\text{ratio}}$ ) between the needle and gelatin tissue phantom over one entire measurement. The control variable was the number of actuation cycles ( C ) set to 15. Table 1 shows the eight experimental conditions evaluated in gelatin phantoms.

**TABLE 1:** Experimental conditions and mean slip ratios for the performance evaluation of the prototype in gelatin phantoms. Experimental condition, central ball spline position fixed or mobile, actuation velocity (rad/s), gelatine weight concentration (wt%), total measured traveled distance (mm) (mean ± standard deviation, *n* = 3), slip ratio (mean ± standard deviation, *n* = 3).

Condition	Central ball spline	Actuation velocity, ω (rad/s)	Gelatin weight concentration (wt%)	Total measured traveled distance, $d_{m}$ (mm) (mean ± std)	Slip ratio, $s_{\text{ratio}}$ (mean ± std)
BF-V1-15%	Fixed	π	15	33 ± 4	0.54 ± 0.052
BF-V2-15%	Fixed	2π	15	26 ± 5	0.64 ± 0.063
BF-V3-15%	Fixed	3π	15	25 ± 1	0.66 ± 0.017
BM-V1-15%	Mobile	π	15	47 ± 3	0.35 ± 0.047
BM-V2-15%	Mobile	2π	15	50 ± 4	0.31 ± 0.060
BM-V3-15%	Mobile	3π	15	51 ± 4	0.29 ± 0.052
BM-V1-10%	Mobile	π	10	58 ± 0	0.19 ± 0.007
BM-V1-5%	Mobile	π	5	59 ± 1	0.18 ± 0.011

### 3.2 Experimental facility and protocol

**FIGURE 7:**
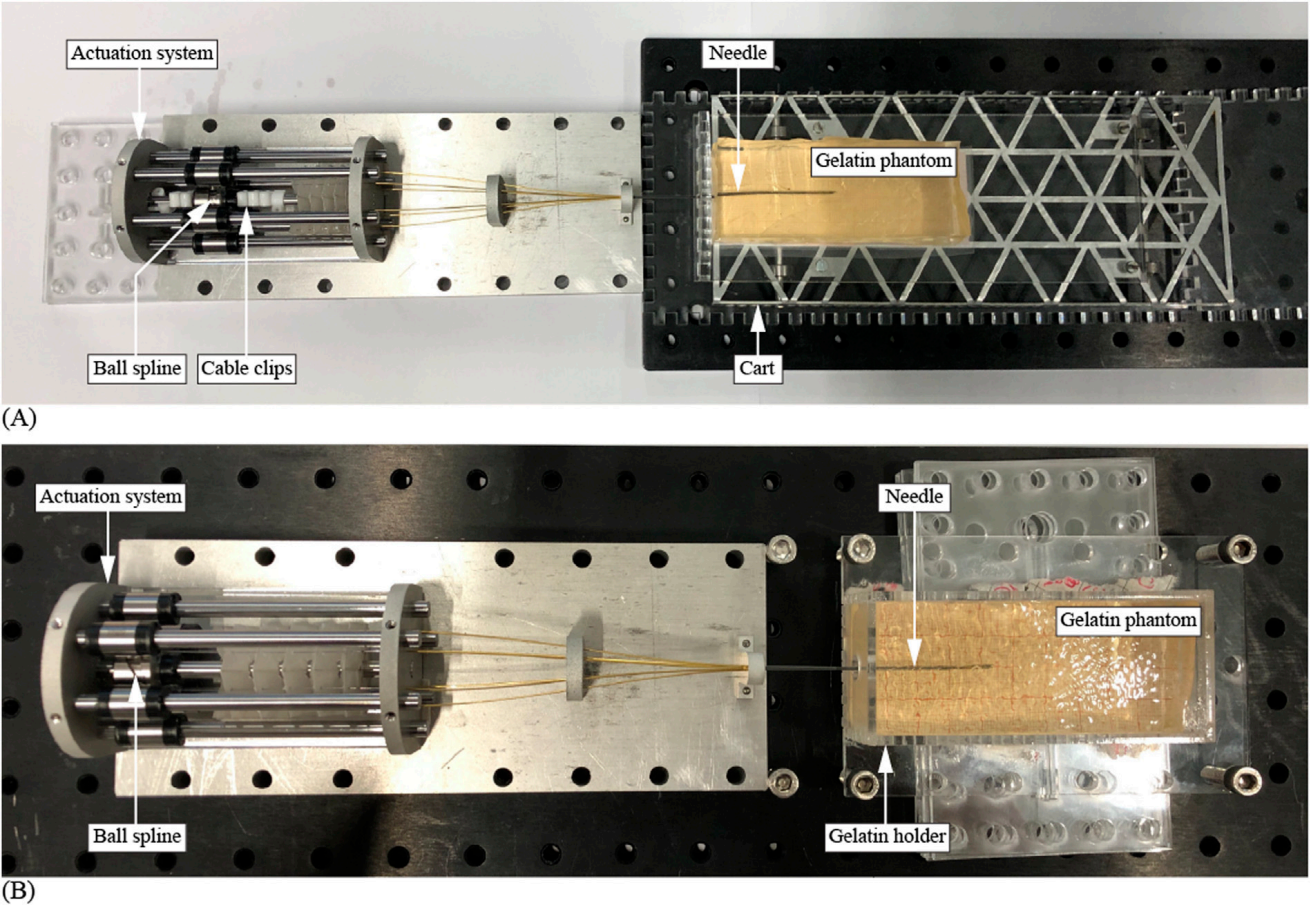
Experimental setup. **(A)** Setup with a fixed ball spline and a mobile gelatin phantom on a low-friction cart. **(B)** Setup with a fixed gelatin phantom and a mobile ball spline mechanism.

The experimental setup with the fixed ball spline differed slightly from the setup with the moving ball spline. The experimental setup with the fixed ball spline consisted of the Splinositor prototype with cable clips to fix the ball spline in place and a gelatin tissue phantom on a low-friction cart (Figure 7A). The experimental setup with the moving ball spline consisted of the prototype and a gelatin tissue phantom contained within a gelatin holder mounted on PMMA plates, which aligned the gelatin holder with the needle, mounted on the breadboard (Figure 7B). The position of the needle tip was recorded using a video camera (iPhone 8) mounted on a tripod, positioned directly above the needle to capture a top-down view of the needle tip within the gelatin phantom. Millimeter graph paper was placed at the bottom surface of the gelatin cart and gelatin holder to give reference to the traveled distance of the needle tip with respect to the gelatin phantom during the experiments with an approximative accuracy of 1 mm. To ensure the repeatability of the measurement method, the experimental setup was not moved in between the measurements.

For the gelatin phantoms, gelatin powder of type *Dr. Oetker 1-50-230004* (Dr. Oetker Professional, Amersfoort, Netherlands) was mixed with water. The gelatin/water mixtures were poured into molds and stored overnight at 5°C to solidify. Subsequently, the gelatin phantoms were cut to their final dimensions, measuring 40 mm in width, 100 mm in length, and 20 mm in height.

For each measurement, a new gelatin phantom was positioned in front of the needle. The needle was inserted over an initial distance of 40 mm to ensure initial contact between the needle segments and the gelatin and to ensure the prototype was inserted in a straight direction. During a single measurement, the Splinositor was actuated for fifteen actuation cycles, i.e., fifteen rotations of the crank. Each experimental condition was repeated three times. After each measurement, we cleaned the needle with water to remove the remaining gelatin.

### 3.3 Results

**FIGURE 8:**
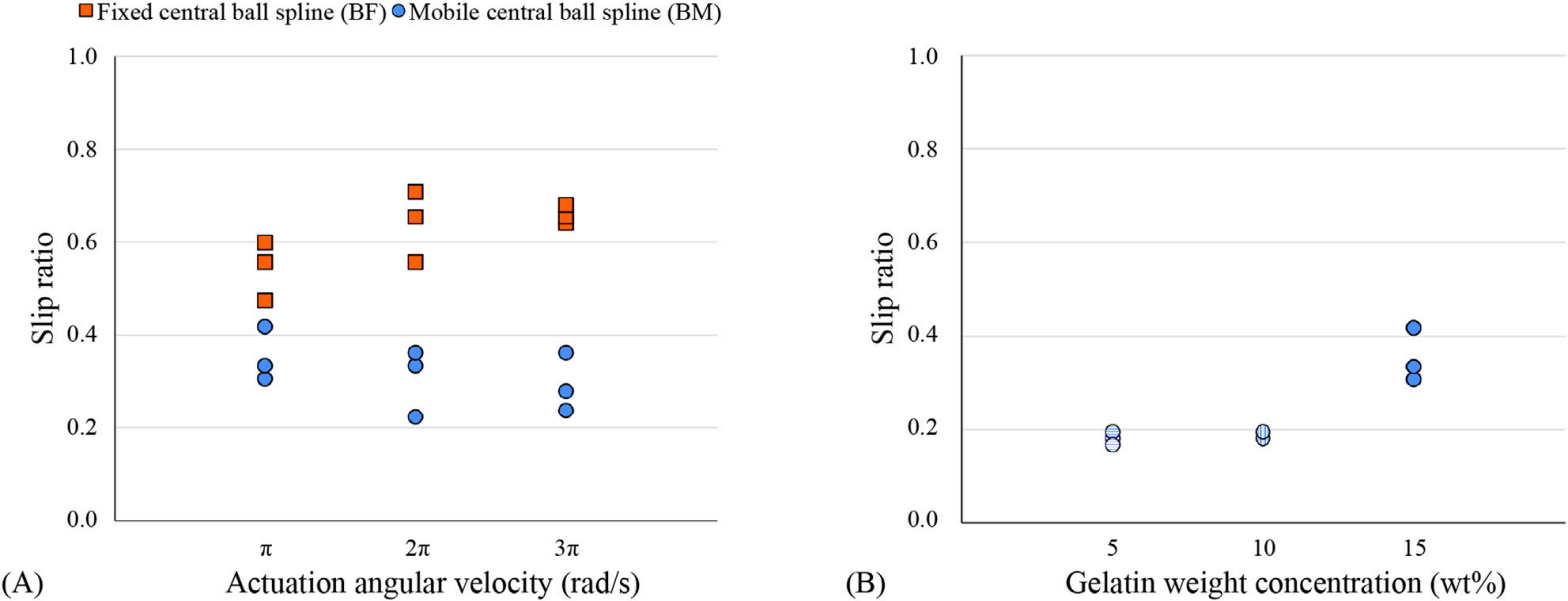
Slip ratio of the needle in gelatin phantoms **(A)** for different actuation angular velocities and **(B)** for different gelatin weight concentrations. The orange squares indicate the single trials for the condition with a fixed base plate, a fixed ball spline, and a mobile gelatin sample on a low-friction cart. The blue circles indicate the single trials for the condition with a fixed base plate, a fixed gelatin sample, and a mobile ball spline.

Table 1 shows the mean and standard deviation of the slip ratio for each experimental condition. Figure 8 shows the slip ratio for each trial. The mean slip ratio in 15-wt% gelatin phantoms was 0.32 for the experimental conditions where the central ball spline was able to move like intended (*n* = 9) and 0.61 for the experimental conditions where the central ball spline was fixed in position and the tissue was placed on a low friction cart (*n* = 9). Also, the mean slip ratio for each velocity evaluated was lower for the conditions where the central ball spline was able to move as intended than for the conditions where the central ball spline was fixed. Increasing the actuation velocity from π to 2π to 3π rad/s resulted in a decrease in the mean slip ratio for conditions where the ball spline was able to move while resulting in an increase in the mean slip ratio for conditions where the ball spline remained fixed. Lastly, decreasing the gelatin weight concentration from 15 wt% to 10 wt% to 5 wt% resulted in a decrease in the mean slip ratio.

## 4 Discussion

### 4.1 Main findings

We have presented the design of a manual actuation system for a needle that uses the self-propelling principle of the female parasitic wasp and can travel through stationary tissue phantoms. The manually controlled actuation system allows the operator to drive the needle directly and intuitively using a continuous manual input rotation. Based on the ball spline mechanism, the actuation system allows the needle to propel through tissue phantoms, while avoiding buckling.

For each actuation velocity evaluated, the slip ratio was higher for the fixed ball spline configuration than that for the movable ball spline. The high slip ratio for the fixed ball spline configuration indicates that the cutting and friction forces acting on the advancing needle segment and the inertia of the tissue cart and friction force acting on the bearings of the tissue cart altogether were near the friction forces on the retracting needle segments (Equation 3). Furthermore, it indicates that the inertia of the tissue cart and friction force acting on the bearings of the tissue cart hinder the self-propelled motion of the wasp-inspired needle more than the inertia and friction force introduced by the components of the actuation system that travel with the needle segments in the positive *y*-direction for the movable ball spline configuration.

The performance evaluation of the Splinositor prototype in the movable ball spline configuration inside 15-wt% gelatin phantoms showed mean slip ratios of 0.35, 0.31, and 0.29 for actuation velocities of π, 2π, and 3π rad/s, respectively. This slip ratio is lower than found in previous research by Scali *et al.* in 15-wt% gelatin phantoms (i.e., slip ratio 0.45–0.8) (Scali et al., 2019), and by Bloemberg *et al.* in *ex-vivo* human prostate tissue (0.86–0.96) (Bloemberg et al., 2022). The mean slip ratios of 0.18 and 0.19 of the Splinositor prototype in the 5-wt% and 10-wt% gelatin phantoms, respectively, are slightly lower than the slip ratios found in previous research by Scali et al. (2019) in 5-wt% and 10-wt% gelatin phantoms (i.e., slip ratios 0.2 and 0.3, respectively). This indicates that the low net insertion force exerted on the needle segments by the actuation system of the Splinositor reduces the slip ratio, thereby enhancing the efficiency of needle advancement through gelatin phantoms, compared to wasp-inspired needles that can self-propel through gelatin phantoms with a zero net insertion force.

### 4.2 Buckling prevention

Previous work regarding wasp-inspired needles shows that a wasp-inspired needle can self-propel through a substrate with a zero net insertion force or even a net pulling force (Frasson et al., 2010; Kataoka et al., 2002). However, to prevent buckling, the insertion force does not need to be zero or negative, rather, it should remain below the critical load of the needle. Therefore, the insertion force exerted on the needle segments by the actuation system should remain below the critical load of the needle segments. When taking the following needle design and tissue parameters: $E_{\text{steel}}=200\text{ GPa}$ , $\emptyset_{\text{needle}\;\text{segment}}=0.3\text{ mm}$ , K=2 for fixed-free end conditions of the needle segment, *L* = 20 mm and 4 mm, which are the maximum unsupported lengths of a needle segment in the actuation system and the substrate, respectively, and μ≈8 kPa for soft tissue (He et al., 2008), the theoretical critical load for each needle segment is approximately 0.5 N. Force measurements of manual insertion of our needle bundle, consisting of six needle segments, into 5-wt% and 10-wt% gelatin phantoms using a force gauge showed peak insertion forces of 0.15 ± 0.082 N (mean ± standard deviation, *n* = 3) and 0.80 ± 0.15 N (mean ± standard deviation, *n* = 3), respectively. This indicates that the insertion force on each needle segment during insertion of the needle bundle in 5-wt% and 10-wt% gelatin samples remains below the critical load of the needle segments. However, in 15-wt% gelatin phantoms, the needle bundle buckled, making it incapable of successful insertion. This indicates that the insertion force on each needle segment required to push the needle bundle into 15-wt% gelatin phantoms was higher than the critical load of the needle segments.

On the other hand, performance evaluation of Splinositor inside 15-wt% gelatin phantoms showed needle insertion without buckling. Therefore, we can conclude that the insertion force exerted on the needle segments by the actuation system remained below the critical load of the needle. We performed force measurements using a force gauge to show the insertion force exerted on the needle segments by the actuation system. The peak force was in the range of 0.05–0.40 N (0.18 ± 0.12 N; mean ± standard deviation, *n* = 5), which indicates the low net insertion force of 0.05–0.40 N remains below the critical load of the needle and therefore prevents needle buckling.

### 4.3 Limitations

The actuation system design in this study allows the needle to travel over 100 mm. This travel distance is limited by the length of the drive cylinder of the ball spline. The follower cylinder of the ball spline and, thus, the cam and the needle segments can travel 100 mm in the *y*-direction. This travel distance can be extended by extending the drive cylinder length, however, this would linearly increase the length of the actuation system design.

The current design consists of a tabletop actuation system and a needle. However, for the design to replace the conventional needles used in percutaneous procedures in a clinical setting, the design should be adapted to a hand-held device. In future prototypes, the actuation system could be miniaturized and included in a handle that moves forward following the wasp-inspired motion of the needle.

The components of the actuation system that travel with the needle segments in the positive *y*-direction have a mass, and therefore, their effect of inertia cannot be ruled out. These components include the follower cylinder of the ball spline, cam, cam followers, and origami structure. To decrease the inertia, the mass of the moving components could be minimized, and the actuation sequence could be adapted to a continuous motion, so the components move with a constant velocity.

Throughout the experiments, we ensured the horizontal alignment of the experimental setup by using a spirit level. This was crucial to prevent gravitational effects on the movement of the follower cylinder of the ball spline and, consequently, the needle segments. In the horizontal position, gravity works solely in the *z*-direction, which is perpendicular to the direction of motion of the needle segments and the corresponding moving components. When the operator tilts the prototype at an angle, creating an inclined plane, the wasp-inspired motion of the needle segments becomes subject to the gravitational forces within the actuation system. In the tilted position, the force of gravity resolves into two components, one parallel to the inclined surface and the other perpendicular to the inclined surface. The force component parallel to the inclined surface would influence the friction forces involved in the wasp-inspired motion.

At the needle tip, the six needle segments are bundled by a heat shrink tube, which might hinder the needle’s propagation into a substrate (e.g., a gelatin phantom or tissue). The needle’s wasp-inspired propulsion with a low net insertion force depends on the surface area of the needle segments in direct contact with the substrate. As the needle is advanced further into the substrate, the surface area of the needle segments in direct contact with the tissue increases, whereas the surface area of the heat shrink tube in contact with the substrate remains unchanged. Consequently, the influence of the heat shrink tube on the needle propulsion declines as the needle advances further into the tissue. Future versions of the Splinositor could incorporate a different bundling mechanism to improve the needle’s wasp-inspired propulsion mechanism.

### 4.4 Recommendations and future research

To assess the prototype’s functioning in a clinical setting, *ex-vivo* or *in-vivo* experiments should be conducted. These experiments will shed light on the effects of inhomogeneous tissue properties and the presence of different tissue layers (e.g., skin, fat, and muscle) with different mechanical properties (e.g., modulus of elasticity), as well as blood, on the self-propelling performance of the needle. The ability of the self-propelled needle to advance in *ex-vivo* human prostate tissue has been exemplified successfully in a previous study (Bloemberg et al., 2022). Furthermore, Scali et al. (2019) showed that the self-propelled needle could advance in multilayered tissue phantoms.

To clinically use the Splinositor as a passageway to a target location inside the body, to inject or extract fluids, extract tissue samples, and position instruments such as radioactive seeds and optical fibers in the body, a functional element should be added as a central element of the needle or should replace one of the needle segments. In future work, it will be interesting to investigate the implementation of a functional element, such as an optical fiber or a tube connected to a syringe, into the Splinositor and investigate the effect on its self-propelled motion. Furthermore, the Splinositor employs a manual actuation system. Further studies could explore alternative actuation methods that allow for downscaling of the actuation system, such as piezoelectric actuation.

In this study, we developed a stationary manual actuation system for a needle that uses the self-propelling principle of the parasitic wasp and can travel through stationary tissue. The moving components of the needle have low inertia. Its connection to the actuation system using a ball spline introduces a small friction force, generating a small insertion force on the needle that facilitates the needle’s propulsion into tissue while preventing needle buckling. In future work, it will be interesting to develop an actuation system capable of exerting an insertion force equivalent in magnitude to the pulling force induced by the wasp-inspired self-propelled motion within the tissue.

## 5 Conclusion

This study presents the design of a manually actuated needle that uses the self-propelling principle of the female parasitic wasp and can travel through stationary tissue phantoms. We have shown that a continuous input rotation can actuate the reciprocating motion of six parallel needle segments using a ball spline-based actuation system. The prototype allows the tissue to remain in place while the needle propels inside the tissue using a low net insertion force of 0.05–0.40 N (0.18 ± 0.12 N; mean ± standard deviation) exerted by the actuation system. The mean slip ratio for each velocity evaluated in 15-wt% gelatin was lower for the conditions where the central ball spline was able to move like intended (i.e., 0.35, 0.31, and 0.29 for π, 2π, and 3π rad/s, respectively) than for the conditions where the central ball spline was fixed (i.e., 0.54, 0.64, and 0.66 for π, 2π, and 3π rad/s, respectively). This indicates that the actuation system’s low net insertion force helps propel the needle through the tissue with a low slip ratio and without buckling. Furthermore, evaluation in 15-wt%, 10-wt%, and 5-wt% gelatin tissue phantoms showed that decreasing the gelatin concentration decreased the mean slip ratios from 0.35 to 0.18 and 0.19, respectively. In conclusion, the ball spline-based actuation system is a step forward in developing a wasp-inspired needle for percutaneous procedures that prevents buckling.

## Data Availability

The original contributions presented in the study are included in the article/Supplementary Material, further inquiries can be directed to the corresponding author.
